# Blood donor exposome and impact of common drugs on red blood cell metabolism

**DOI:** 10.1172/jci.insight.146175

**Published:** 2021-02-08

**Authors:** Travis Nemkov, Davide Stefanoni, Aarash Bordbar, Aaron Issaian, Bernhard O. Palsson, Larry J. Dumont, Ariel Hay, Anren Song, Yang Xia, Jasmina S. Redzic, Elan Z. Eisenmesser, James C. Zimring, Steve Kleinman, Kirk C. Hansen, Michael P. Busch, Angelo D’Alessandro, the Recipient Epidemiology and Donor Evaluation Study III Red Blood Cell–Omics (REDS-III RBC-Omics) Study

**Affiliations:** 1 Department of Biochemistry and Molecular Genetics, University of Colorado Denver — Anschutz Medical Campus, Aurora, Colorado, USA.; 2 Omix Technologies Inc., Aurora, Colorado, USA.; 3 Sinopia Biosciences Inc., San Diego, California, USA.; 4 University of California San Diego, San Diego, California, USA.; 5 Vitalant Research Institute, Denver, Colorado, USA.; 6 University of Virginia, Charlottesville, Virginia, USA.; 7 University of Texas Health Science Center at Houston, Houston, Texas, USA.; 8 University of British Columbia, Victoria, British Columbia, Canada.; 9 Vitalant Research Institute, San Francisco, California, USA.; 10 The REDS-III RBC–Omics Study is detailed in Supplemental Acknowledgments.

**Keywords:** Hematology, Metabolism, Drug screens

## Abstract

Computational models based on recent maps of the RBC proteome suggest that mature erythrocytes may harbor targets for common drugs. This prediction is relevant to RBC storage in the blood bank, in which the impact of small molecule drugs or other xenometabolites deriving from dietary, iatrogenic, or environmental exposures (“exposome”) may alter erythrocyte energy and redox metabolism and, in so doing, affect red cell storage quality and posttransfusion efficacy. To test this prediction, here we provide a comprehensive characterization of the blood donor exposome, including the detection of common prescription and over-the-counter drugs in blood units donated by 250 healthy volunteers in the Recipient Epidemiology and Donor Evaluation Study III Red Blood Cell–Omics (REDS-III RBC-Omics) Study. Based on high-throughput drug screenings of 1366 FDA-approved drugs, we report that approximately 65% of the tested drugs had an impact on erythrocyte metabolism. Machine learning models built using metabolites as predictors were able to accurately predict drugs for several drug classes/targets (bisphosphonates, anticholinergics, calcium channel blockers, adrenergics, proton pump inhibitors, antimetabolites, selective serotonin reuptake inhibitors, and mTOR), suggesting that these drugs have a direct, conserved, and substantial impact on erythrocyte metabolism. As a proof of principle, here we show that the antacid ranitidine — though rarely detected in the blood donor population — has a strong effect on RBC markers of storage quality in vitro. We thus show that supplementation of blood units stored in bags with ranitidine could — through mechanisms involving sphingosine 1–phosphate–dependent modulation of erythrocyte glycolysis and/or direct binding to hemoglobin — improve erythrocyte metabolism and storage quality.

## Graphical abstract

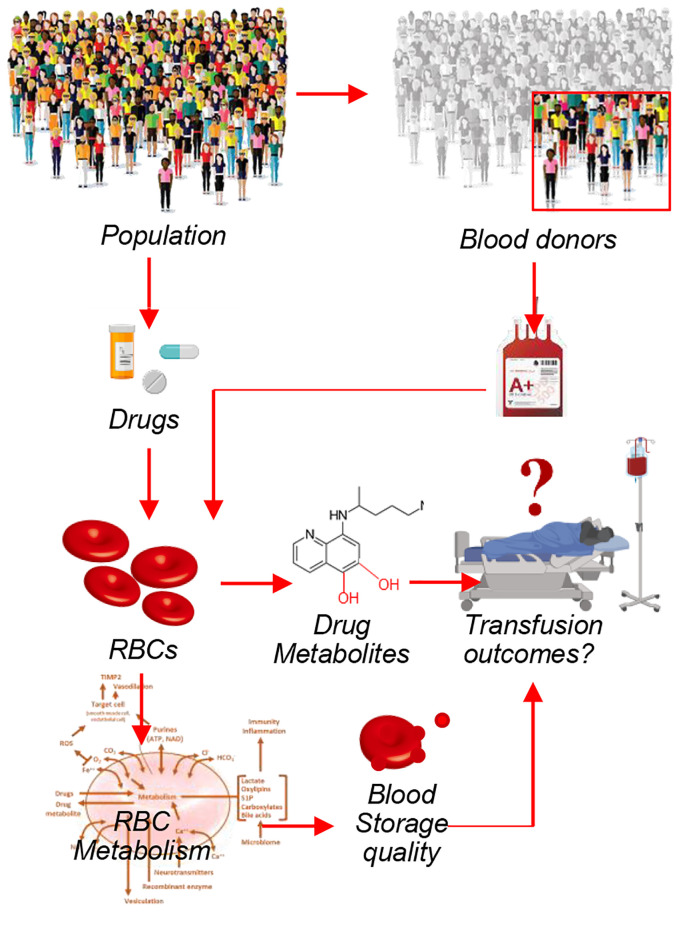

## Introduction

Despite being the most abundant cell type in the human body (1), RBCs are usually regarded as a bystander in systems physiology. RBCs play a critical role in the transport and delivery of oxygen to tissues, a task that they have evolved to fulfill by maximizing the oxygen-carrying capacity: having lost nuclei and organelles, the mature RBC carries approximately 250–270 million molecules of hemoglobin per cell (2). Thus, most textbooks refer to the mature erythrocyte as a mere circulating bag of hemoglobin that serves no other purpose. Two decades of advances in the field of proteomics have revealed that while approximately 90% of the dry weight of a mature erythrocyte is indeed composed of hemoglobin, the remaining 10% harbors a rich and diverse protein machinery of approximately 3000 gene products (3). Through this machinery the mature RBC is capable of responding to environmental stimuli (e.g., hypoxia and oxidant stress) by mechanisms that involve posttranslational modifications (especially phosphorylation, ref. 4; and methylation, ref. 5) and metabolic reprogramming. These cascades are usually triggered by direct stimulation of receptors on the cell surface (e.g., adenosine receptor in response to high-altitude hypoxia; ref. 6) or hemoglobin-oxygen saturation, which affects the compartmentalization and activity of several metabolic and antioxidant enzymes, such as glyceraldehyde 3-phosphate dehydrogenase (7, 8) and peroxiredoxin 2 (9), respectively. These mechanisms of oxygen-dependent metabolic modulation also promote a feedback in energy and redox metabolism, closing a loop that ultimately affects the capacity of RBCs to capture, carry, and off-load oxygen. The receptors include purinergic, muscarinic, dopaminergic, and other neurotransmitter-stimulated receptors (10), making the mature RBC theoretically sensitive to small molecules that act as agonist or antagonist of those signaling pathways in a way that could ultimately affect RBC metabolism and, in so doing, hemoglobin capacity to bind and off-load oxygen.

The unanticipated complexity of the RBC proteome suggests that the mature erythrocyte could not only respond upon stimulation of specific drug targets — in silico predictions suggest that approximately 230 targets to common drugs may exist in the mature RBC (11) — but also theoretically participate in drug metabolism. Some of the proteins compiled in the latest proteomics studies (3, 12), if functional, could play a role in cellular — and maybe systems — metabolism. For example, recent studies have shown that RBCs are endowed with several transporters, as well as diaphorases and enzymes with cytochrome *b*_5_ reductase activity (13), which allows them to metabolize antimalarial drugs such as chloroquine (14) or antiretroviral drugs (e.g., tenofovir; ref. 15).

Understanding the impact of xenometabolites (including drugs) on RBC metabolism and biology may hold key implications in the field of transfusion medicine, since RBC transfusion — a lifesaving intervention for millions of people worldwide — is the most common iatrogenic intervention after vaccination. While blood storage is a logistic necessity for making more than 100 million units available for transfusion every year worldwide, refrigerated storage in the blood bank is accompanied by the progressive accumulation of storage lesions (16). In recent years, the introduction of omics technologies in the field of transfusion medicine has expanded our understanding of the mechanisms underlying the progression and severity of the storage lesion (17). Oxidant stress to metabolic enzymes, and to structural and functional proteins (5, 8, 18) ultimately results in RBCs that are more susceptible to lysis in the bag or untimely removal from the bloodstream in the recipient. The rate at which the storage lesions occur and the extent to which these phenomena progress by the end of the shelf-life of a blood unit (42 days in the United States) vary depending on processing strategies, storage bags and solutions, and donor biology (19–21). These factors have emerged clearly from the Recipient Epidemiology and Donor Evaluation Study (REDS-III) RBC-Omics Study, in which 13,403 healthy whole blood donor volunteers consented to participate in a large-scale assessment of donor-dependent factors on RBC storability and hemolytic propensity at the end of storage. In the mature RBC, additional factors such as sex, ethnicity, age (20), donation frequency, and genetic polymorphisms are associated with a decreased capacity to cope with osmotic and oxidant stress (e.g., glucose 6–phosphate dehydrogenase deficiency) and ultimately affect the metabolic age and posttransfusion efficacy of the unit (22, 23).

It is a logical consequence of the studies described above that the antioxidant capacity of a mature RBC is affected by other factors beyond donor biology that could counteract or aggravate oxidant stress. One such factor is represented by donor habits, such as smoking, which in smaller cohorts has been shown to increase markers of oxidant stress in the stored unit (e.g., carboxyhemoglobin or altered glutathione homeostasis; ref. 24) and ultimately result in lower hemoglobin increments in recipients of units from smoking donors (25). However, it is unclear whether and to what extent other factors derived from environmental exposures — referred to as the “exposome”(26) — would impact the metabolic age of the stored RBC and, as a result, transfusion efficacy in the recipient.

## Results

### Identification of xenometabolites in the blood of healthy donor volunteers.

**Figure 1:**
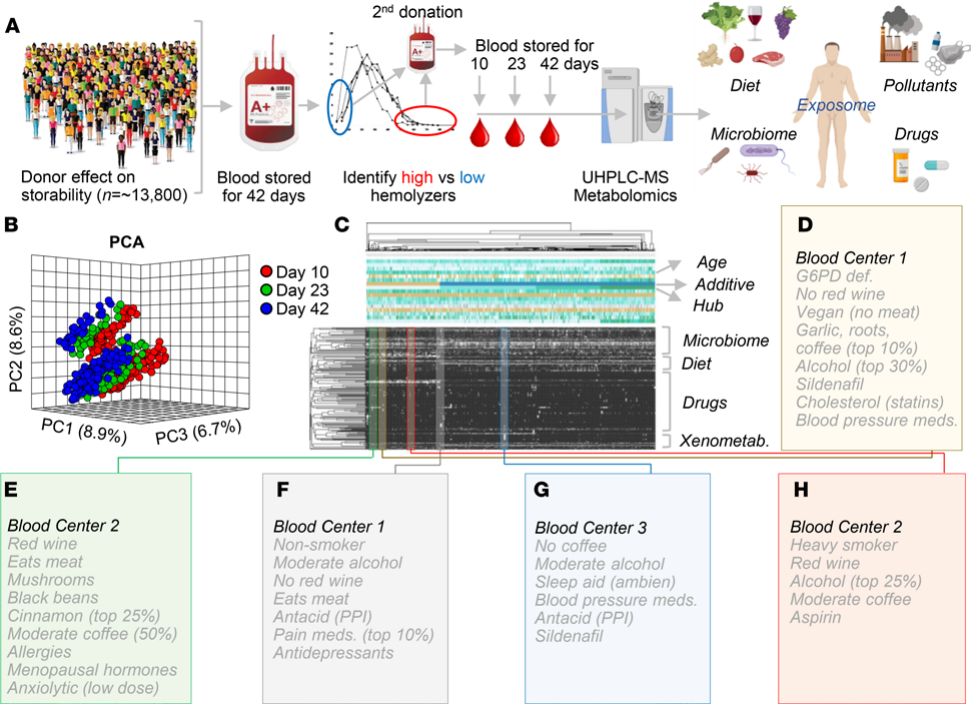
RBC exposome in the REDS-III RBC-Omics Study. (**A**) Within the framework of the REDS-III RBC-Omics Study, 13,800 healthy donor volunteers were enrolled to donate a unit of whole blood that was processed into leukoreduced erythrocyte concentrates. Units were stored until the end of their shelf life (42 days), when they were tested for the propensity of RBCs to hemolyze, spontaneously or following osmotic and oxidative stress. Donors in the 5th and 95th percentiles for hemolysis measurements were asked to donate a second unit of blood, which was sampled on storage days 10, 23, and 42 for metabolomics analyses of the RBC exposome, i.e., the compendium of small molecule metabolites derived from diet, the microbiome, or other xenometabolites, such as pollutants or drugs. (**B**) Unsupervised PCA of the exposome data clustered the subjects (dots) on the basis of storage duration (red, green, and blue for days 10, 23 and 42, respectively). (**C**) Hierarchical clustering analysis of blood donors (*n* = 250) on storage day 10 on the basis of exposome measurements (black to white: absent to high-abundance). (**D**–**H**) The exposome informs on donor metabolic status and various habits, as highlighted in a few cases from the hierarchical clustering analysis. PPI, proton pump inhibitor; def., deficiency.

**Figure 2:**
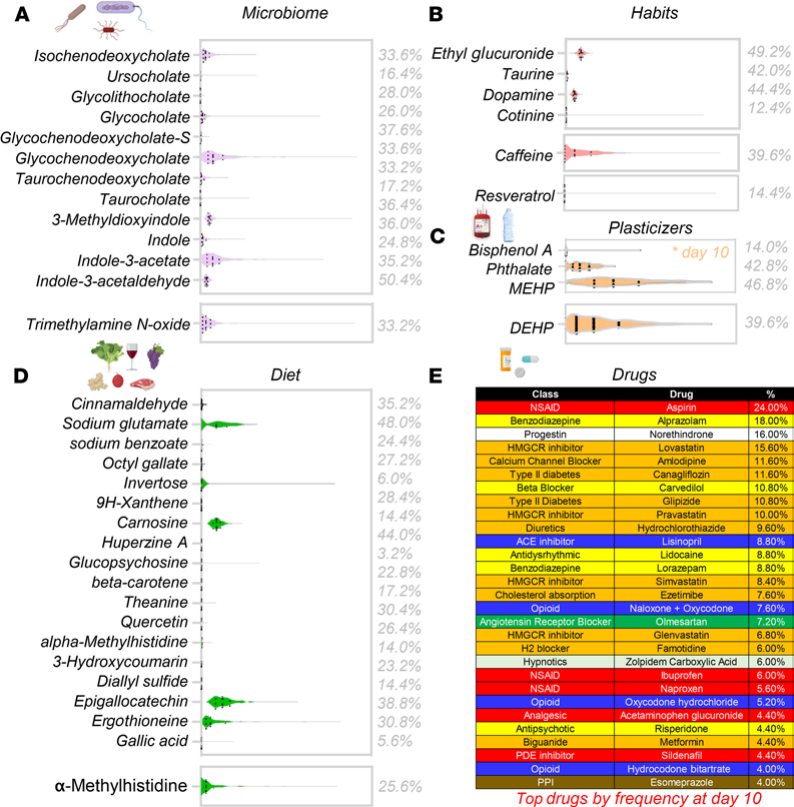
RBC exposome in the REDS-III RBC-Omics Study. (**A**–**E**) Overview of the most abundant exposome metabolites of microbial origin, habits (drinking alcohol or caffeinated beverages; nicotine exposure), plasticizers, dietary metabolites, or common drugs (with percentage of day 10 stored blood donor samples in which traces of those drugs were detected).

**Figure 3:**
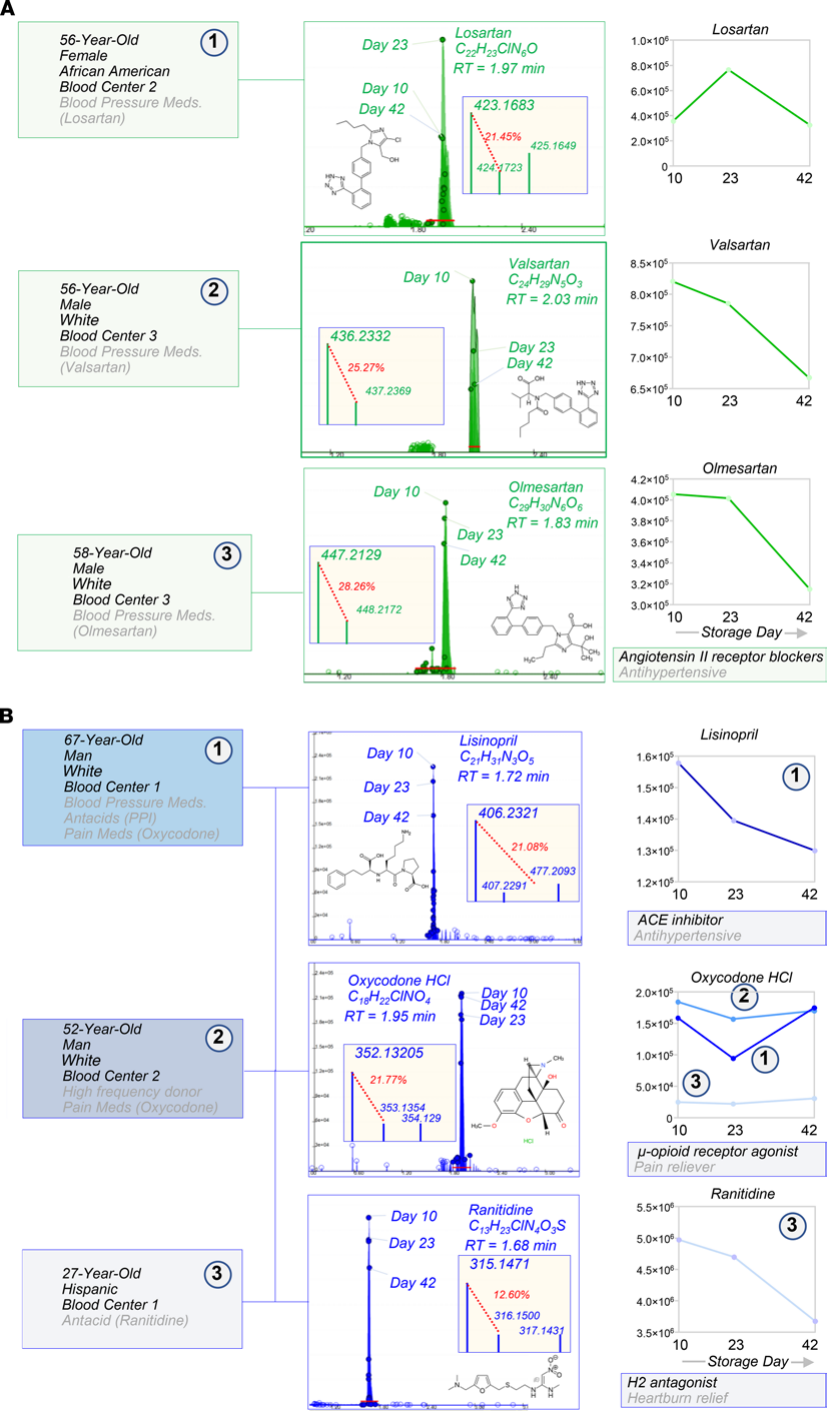
Exposome: traces of sartans, antihypertensive drugs, and pain medications were detected in a small subset of RBCs from healthy donor volunteers. Case studies are described in which the drugs were detected included sartans (green, **A**), antihypertensive drugs, and pain medications (blue, **B**). For each metabolite, we provide the original extract ion chromatograms on the background of the whole population (599 samples), molecular formulas, retention times (RT), mass spectra, ^13^C abundance as percentage of the parent *m/z* peak, and time course levels (*y* axes indicate integrated peak areas [AU]).

**Figure 4:**
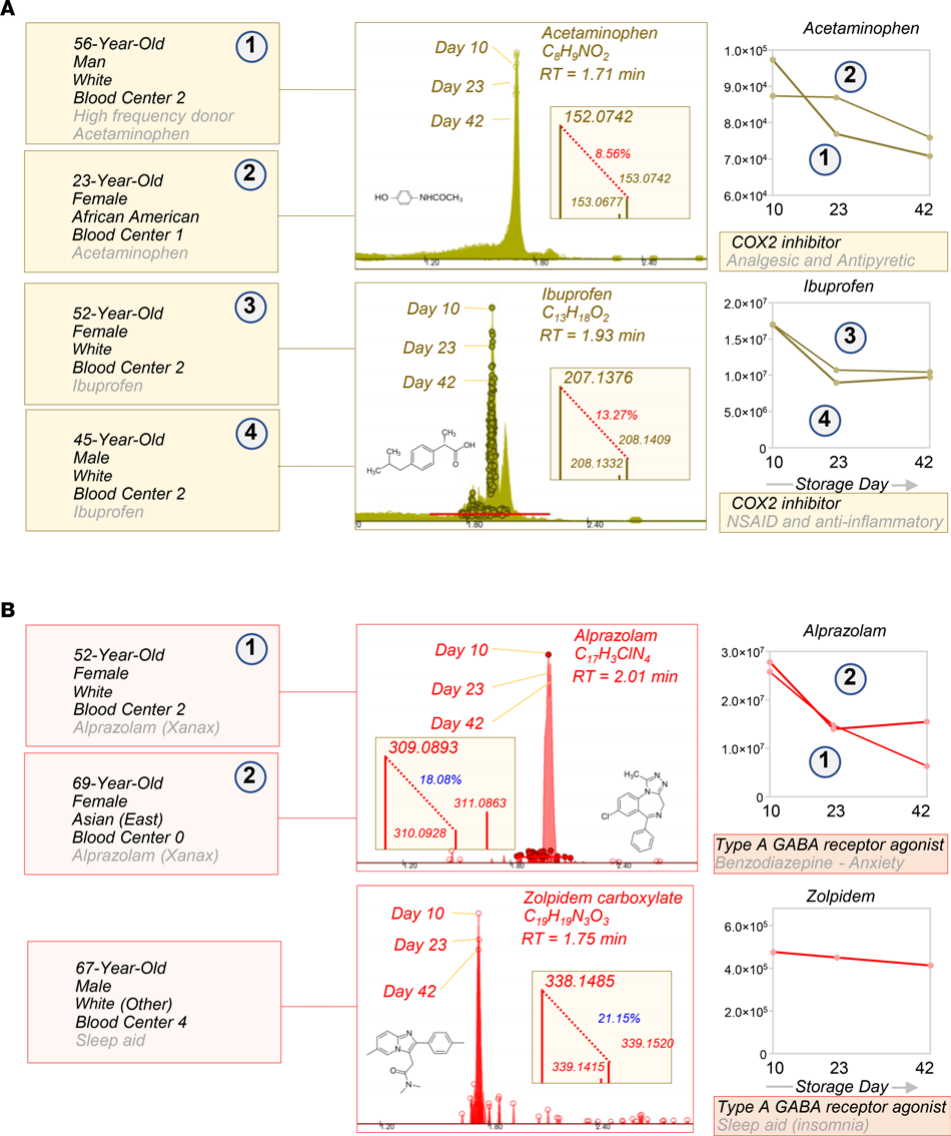
Exposome: traces of antiinflammatory drugs, antidepressants, and sleep aids were detected in a small subset of RBCs from healthy donor volunteers. Case studies are described in which NSAIDs and other antiinflammatory drugs (gold, **A**), antidepressants, and sleep aids (red, **B**) were detected. For each metabolite, we provide the original extract ion chromatograms on the background of the whole population (599 samples), molecular formulas, retention times, mass spectra, ^13^C abundance as percentage of the parent *m/z* peak, and time course levels (*y* axes indicate integrated peak areas [AU]).

Blood units from 250 healthy donor volunteers were sampled at storage days 10, 23, and 42 for metabolomics analyses of the RBC exposome, i.e., the compendium of small molecule metabolites derived from diet, the microbiome, or other xenometabolites, such as pollutants or drugs (ref. 26, Figure 1A, and data in Supplemental Table 1; supplemental material available online with this article; https://doi.org/10.1172/jci.insight.146175DS1). To ensure comprehensiveness of the analysis, a custom database was imported from the Blood Exposome Database website (https://bloodexposome.org/) (Supplemental Table 1 and ref. 27). Unsupervised principal component analyses (PCA) clustered the subjects on the basis of storage duration (red, green, and blue for days 10, 23, and 42, respectively — across PC1) and additive solution (across PC2; Figure 1B). Hierarchical clustering analysis of the exposome data (Figure 1C) highlighted groups of donors with unique metabolic status and various habits (Figure 1, D–H). Dot plots in Figure 2, A–D, provide an overview of the levels of the most abundant exposome metabolites of potential microbial origin (such as indoles generated by the gut microbiome or conjugated bile acids that are deconjugated exclusively by gut bacteria; ref. 28), derived from donor habits (e.g., drinking alcohol or caffeinated beverages; nicotine exposure), plasticizers (e.g., from blood bags), and dietary metabolites. Of note, when searching against a recent list of the most common prescription or over-the-counter drugs in the United States (29), we could detect traces of most compounds in at least one donor — with the most frequently detected compounds listed in Figure 2E (full report in Supplemental Table 1). For example, traces of drugs detected in RBC units included angiotensin II receptor inhibitors, NSAIDs and other antiinflammatory drugs, antihypertensives, pain medications, antidepressants, and sleep aids (Figures 3 and 4); and drugs targeting specific metabolic enzymes, such as the rate-limiting enzyme in cholesterol synthesis — hydroxymethyl-glutaryl CoA reductase inhibitors, in the case of statins — or systems metabolism at large (e.g., biguanides such as metformin) (Supplemental Figure 1). Correlation of exposome metabolites to spontaneous, oxidative, osmotic, and mechanical fragility highlighted some interesting trends (Supplemental Table 1). For example, 3 units from subjects older than 70 years tested positive for sildenafil — an inhibitor of the enzyme phosphodiesterase E5 that regulates cGMP metabolism — and its catabolite desmethyl sildenafil (Supplemental Figure 2). Sildenafil was associated with increased level of oxidative stress–related metabolites and increased susceptibility to oxidant stress–induced hemolysis, but lower osmotic fragility, as gleaned by unsupervised PCA, hierarchical clustering, and pathway analyses (Supplemental Figure 2, E–G).

Among all the units that tested positive for any drugs, the most significant deviation from the energy and redox metabolic phenotypes of the general population were noted in samples from subjects who tested positive for cholesterol medications (statins), blood pressure medications (angiotensin II receptor blockers), and antacids (such as proton-pump inhibitors or antihistamine drugs; Supplemental Figure 3).

### High-throughput metabolomics confirms a direct impact of FDA-approved drugs on RBC metabolism.

**Figure 5:**
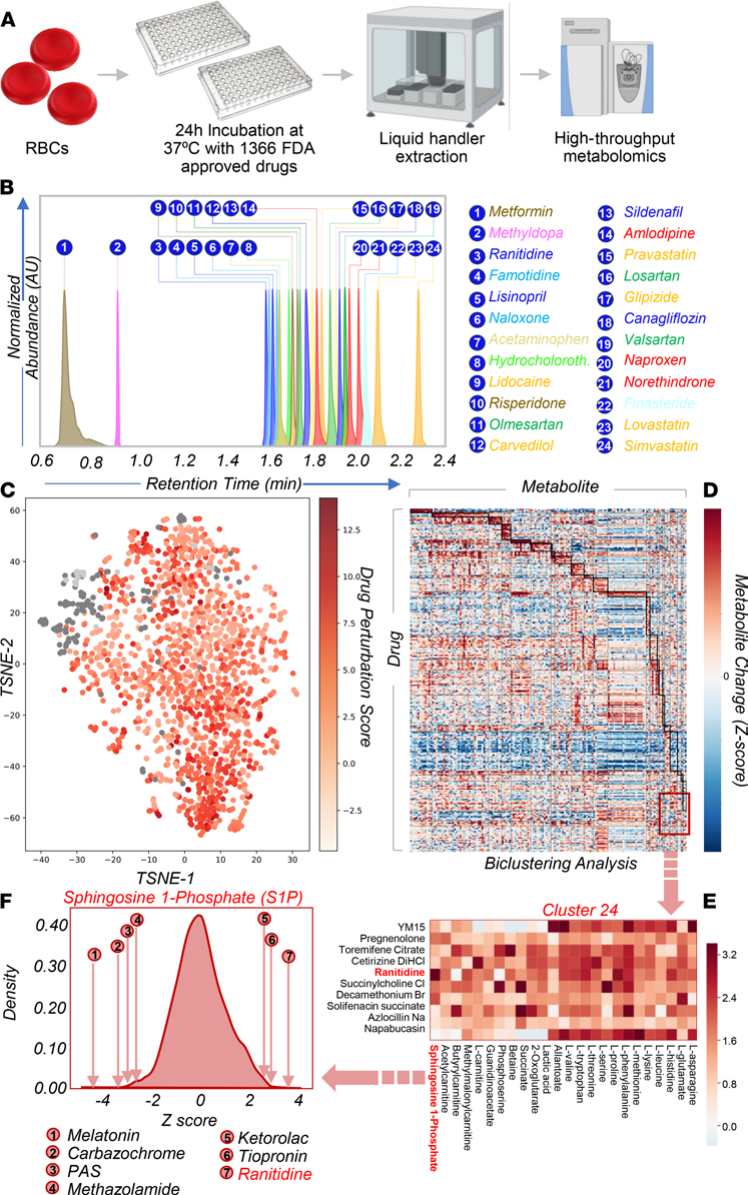
High-throughput metabolomics screening of human RBCs. (**A**) Prior to high-throughput metabolomics screening, donor RBCs were incubated with 1366 FDA-approved drugs for 24 hours at 37°C. (**B**) Retention times for most commonly identified drugs in the REDS-III cohort were validated in the high-throughput screening (*y* scale is in AU, normalized across all drugs). Hydrochloroth., hydrochlorothiazide. (**C**) TSNE plot of 1507 metabolomics samples of erythrocytes exposed to small molecule compounds (1368 samples), treated with vehicle (121 samples, dark gray), or left untreated (18 samples, light gray). Vehicle-treated and untreated samples cluster together. Though there is not a clear clustering of small molecule compounds into specific groups, they generally cluster by drug perturbation *z* score. (**D**) Heat map of subset of the metabolomics data of erythrocytes exposed to small molecules. Biclustering was used to identify modules, which are sets of drugs and set of metabolites whose changes significantly correlate with each other. Modules are shown in dark black boxes. Twenty-five total modules were identified due to perturbations by 331 small molecules affecting 151 metabolites. Identified modules and associated small molecules and metabolites are provided in Supplemental Table 1. (**E**) Highlight of metabolites and drug in cluster 24 from the biclustering analysis. (**F**) Effects of small molecule drugs from the high-throughput screening on the metabolite S1P (one of the metabolites most significantly affected in cluster 24). Four compounds that decrease S1P the most and 3 drugs that increase S1P the most are shown. Melatonin is known to have an effect on the S1P signaling pathway. Carbazochrome is a hemostatic agent. PAS is an antibiotic for tuberculosis but is contraindicated in individuals with G6PDH deficiency, as it induced hemolysis. Methazolamide is a carbonic anhydrase inhibitor. Ranitidine is an antihistamine; histamine is known to affect S1P signaling.

In order to systematically validate the putative identifications and understand the potential impact of FDA-approved drugs on donor units, we performed a high-throughput metabolomics screening of human RBCs incubated with a panel of 1366 FDA-approved drugs for 24 hours at 37°C (Figure 5, A and B). To determine the metabolic impact of (classes of) drugs on RBCs, we calculated t-distributed stochastic neighbor embedding (TSNE) plots for 1505 metabolomics samples of erythrocytes exposed to small molecule compounds (1366 samples) or vehicle (121 samples) or left untreated (18 samples; Figure 5C). Upon normalization (Supplemental Figure 4), unsupervised analyses showed that vehicle-treated and untreated samples clustered together. While we did not observe a clear clustering of small molecule compounds into specific classes, samples generally clustered by the magnitude of the drug perturbation (Figure 5C). Indeed, small molecule drugs were found to alter the levels of erythrocyte metabolites that have been implicated in several pathologies and storage-related changes (17), including reduced glutathione, lactic acid, *S*-adenosyl-l-methionine, and hypoxanthine (Supplemental Figure 5). In addition, machine learning models built using metabolites as predictors were able to accurately predict drugs for several drug classes/targets (i.e., bisphosphonates, anticholinergics, calcium channel blockers, adrenergics, proton pump inhibitors, antimetabolites, selective serotonin reuptake inhibitors, and mTOR), suggesting that these drugs have a direct, conserved, and significant impact on RBC metabolism (Supplemental Table 1). To further understand the impact on RBC metabolism, we used biclustering to identify “modules” (Supplemental Table 1), which are sets of drugs that induce similar changes to a similar set of metabolites. A total of 25 significant modules were identified due to perturbations by 331 small molecules affecting 151 metabolites (Figure 5D and Supplemental Table 1). Of note, we also observed that RBC can metabolize a subset of the 1366 drugs with which they were incubated in the high-throughput screening, including primaquine to primaquine *N*-acetate and 5-hydroxy-desmethyl-primaquine (active metabolite; Supplemental Figure 6A); tenofovir disoproxil to tenofovir metabolites (Supplemental Figure 6B); and doxorubicin to doxorubicinol (Supplemental Figure 6C).

### Ranitidine promotes increases in RBC sphingosine 1–phosphate.

To validate some of the findings from the high-throughput screening, we focused on cluster 24 from the biclustering analysis (Figure 5E). This cluster included drugs with a significant effect on RBC markers of storage quality, as determined in our previous studies (30), e.g., lactic acid, a marker of glycolysis — the main energy pathway in RBCs; acyl-carnitines, markers of membrane lipid homeostasis (31) and propensity to hemolyze following osmotic insults (32); amino acids, markers of ion and protein homeostasis (10); carboxylic acids (2-oxoglutarate and succinate), markers of altered homeostasis of reducing equivalents (33); and sphingosine 1–phosphate (S1P), a major regulator of RBC glycolysis and function (i.e., oxygen off-loading) under physiological and pathological conditions (34–36). To illustrate the rationale behind the selection of a drug with the greatest effects on the above-mentioned metabolites, in Figure 5F we show that a subset of drugs were found to significantly decrease (e.g., melatonin, carbazochrome, 4-aminosalicylic acid [PAS], methazolamide) or increase (ranitidine, tiopronin and ketorolac) RBC S1P levels by more than 4 standard deviations from the mean (*z* score–normalized values). Interestingly, melatonin has been reported to inhibit sphingosine kinase 1 (Sphk1, ref. 37) — the rate-limiting enzyme of S1P synthesis in RBCs. Ranitidine is an antihistamine (histamine H2 receptor antagonist), which is relevant in that histamine has been reported to positively regulate Sphk1 activity (38). We thus predicted that the antacid ranitidine, which we had previously found to affect metabolic markers of RBC storage lesions (30) — subsequently validated as predictors of hemolysis and posttransfusion recovery (the FDA gold standard for blood storage; ref. 39) —could improve RBC storage quality.

### Detection of ranitidine is associated with elevated S1P metabolism and S1P-regulated glycolysis in stored RBCs from the REDS-III RBC-Omics study.

**Figure 6:**
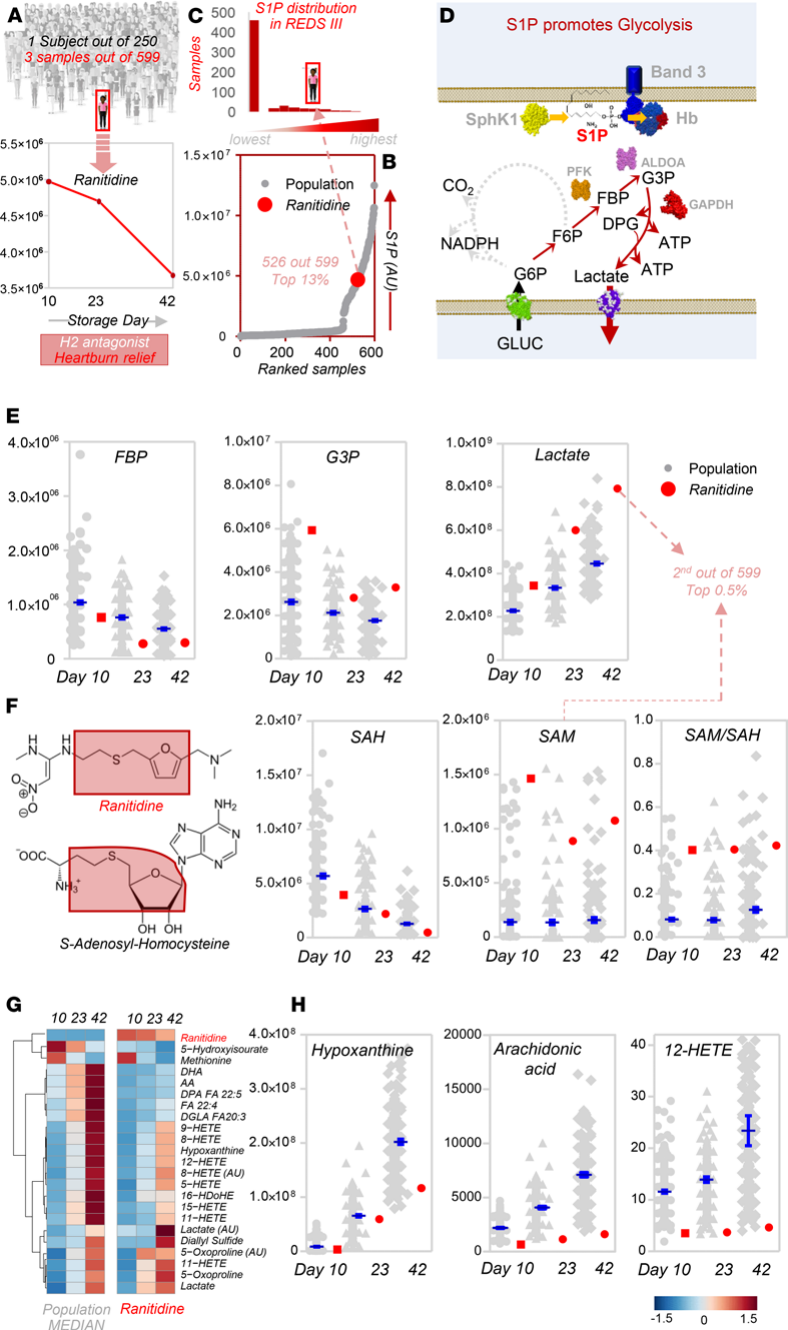
Ranitidine impacts S1P metabolism and S1P-regulated glycolysis in stored RBCs from the REDS-III RBC-Omics Study. (**A**) Only one subject was identified to be positive for ranitidine among 599 samples from 250 independent donors in the recalled REDS-III RBC-Omics donor cohort. (**B** and **C**) S1P levels in RBCs from this subject on storage days 10, 23, and 42 ranked in the top 13% among all 599 samples tested in this study. (**D**) Schematic representation of the mechanisms through which S1P levels in RBCs modulate glycolysis, as previously described (34). (**E**) Consistently, faster consumption of early glycolytic intermediates (fructose bisphosphate [FBP]) and increased levels of glyceraldehyde 3-phosphate (G3P) and the final byproduct of glycolysis, lactate, were detected in the subject positive for ranitidine, with end-of-storage lactate levels ranking second among all the 599 samples tested (top 0.5%). (**F**) Given the structural similarity between ranitidine and SAH, we hypothesized and confirmed an interaction of this metabolite with the levels of SAH and related metabolites (SAM and SAM/SAH ratios). (**G**) Heat map of the top 25 metabolites that differ significantly between the subject positive for ranitidine on any storage day (10, 23, and 42) and the median of the rest of the population. (**H**) The map highlighted that several metabolic markers of the RBC storage lesion are decreased in the stored RBCs from this subject, including markers of posttransfusion recovery hypoxanthine, arachidonic acid, and 12-HETE. In **E**, **F**, and **H**, *y* axes indicate integrated peak areas (in AU), except for SAM/SAH (ratios between the 2 metabolites) and arachidonic acid and 12-HETE (nM).

First, we set out to determine whether ranitidine could be detected in donor blood from the population enrolled in this study and, if so, whether the metabolic phenotype of ranitidine-positive units recapitulated the beneficial metabolic effects observed in the high-throughput screening. With respect to the former question, only one subject tested positive for ranitidine in the recalled REDS-III RBC-Omics donor cohort (Figure 6A). However, samples from this donor ranked in the top 13% S1P levels of all the 599 samples tested (Figure 6, B and C). In previous studies, we had shown that RBC S1P promotes glycolysis by stabilizing the tense deoxygenated state of hemoglobin, which in turn outcompetes glycolytic enzymes that are otherwise bound to and inhibited by the N-terminus of band 3 (model in Figure 6D and refs. 34–36). Consistently, RBCs from the ranitidine-positive unit were characterized by end-of-storage lactate levels in this subject that ranked second among all 599 samples tested in this study (Figure 6E). Given the structural similarity between ranitidine and *S*-adenosyl-homocysteine (SAH; Figure 6F), here we hypothesized and observed an association between the detection of ranitidine and the levels of SAH and related metabolites (*S*-adenosylmethionine [SAM] and SAM/SAH ratios; Figure 6F), metabolites critical in oxidant stress–induced isoaspartyl protein damage repair in the stored erythrocyte (5). Consistent with our prediction based on the high-throughput screening, RBCs from the subject on ranitidine were characterized by decreases in levels of several metabolic markers of the storage lesion (Figure 6G), including the markers of poor posttransfusion recovery hypoxanthine, arachidonic acid, and 12-hydroxyeicosatetraenoic acid (12-HETE) (lowest levels among all 250 subjects enrolled in the study; Figure 6H and refs. 30, 40).

### Ranitidine boosts S1P levels and glycolysis in a dose-response fashion in human and WT mouse RBCs but not in Sphk1-KO mice.

**Figure 7:**
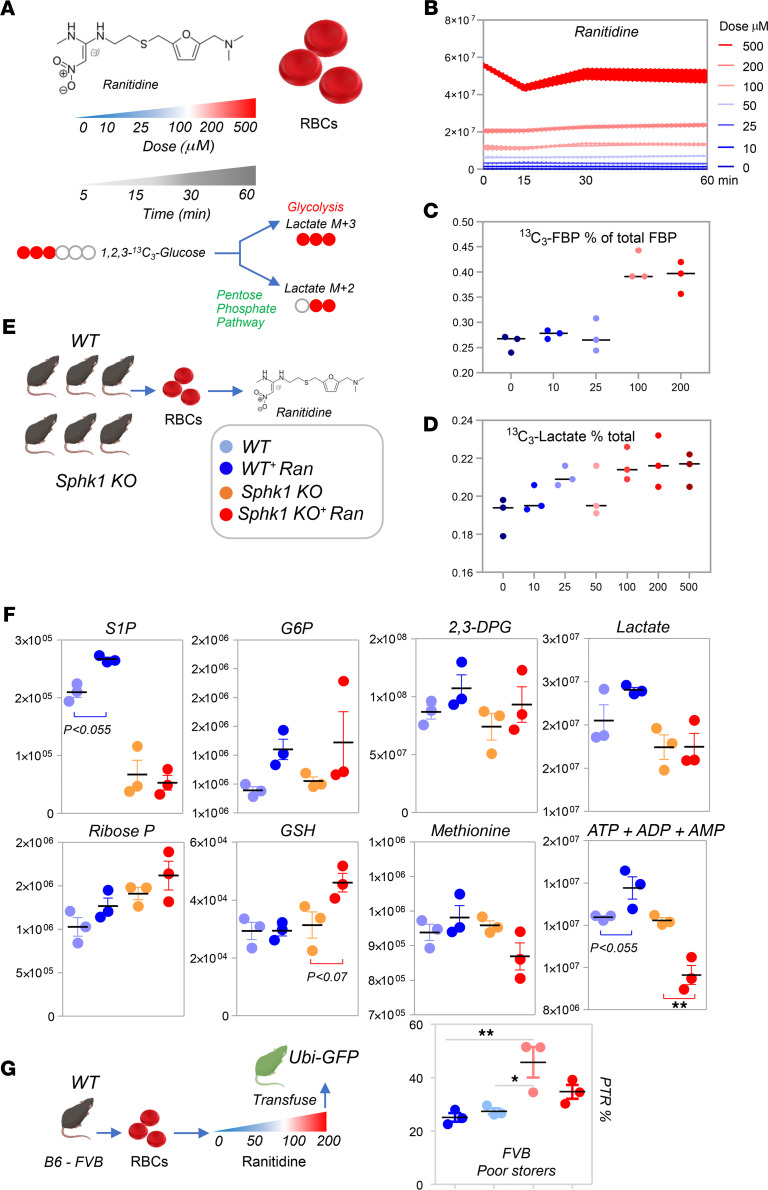
Ranitidine boosts glycolysis in a dose-response fashion in human RBCs. (**A**) Human RBCs (*n* = 3) were incubated with increasing doses of ranitidine in the presence of 1,2,3-^13^C_3_-glucose for up to 60 minutes. At increasing doses of ranitidine (**B**) corresponded increasing levels of ^13^C_3_-labeled 1,6-fructose diphosphate (rate-limiting step of glycolysis, plateauing at 100 μM ranitidine; **C**) and ^13^C_3_-lactate (**D**) — suggestive of increased fluxes through glycolysis but not the Rapoport-Luebering shunt. (**E**) RBCs were also obtained from 3 WT and 3 Sphk1-KO mice, prior to incubation with 100 μM ranitidine for 60 minutes. (**F**) After confirming significant decreases in S1P levels in the Sphk1-KO mouse RBCs, we observed that ex vivo incubation with ranitidine promoted increases in the levels of several glycolytic intermediates, including G6P, G3P, 2,3-DPG, phosphoglycerate, and pyruvate. However, increases in the total levels of lactate were observed in WT but not in Sphk1-KO mice following incubation with ranitidine. On the other hand, Sphk1-KO mice showed increased steady-state levels of ribose phosphate, suggestive of increased fluxes through the pentose phosphate pathway and increased NADPH-dependent recycling of glutathione. This observation is consistent with the observed increases in levels of reduced glutathione in Sphk1-KO mice after incubation with ranitidine. These mice were characterized by lower levels of another antioxidant, methionine. (**F**) Finally, the total adenylate pool (high-energy phosphate compounds including adenosine tri-, di-, and monophosphate [ATP + ADP + AMP]) increased in WT cells following incubation with ranitidine, but decreased in Sphk1-KO mouse RBCs. Color key in **E** applies to **E** and **F**. (**G**) Storage of RBCs from mice with poor storage quality (FVB, *n =* 3) in the presence of ranitidine (0, 50, 100, or 200 μM) resulted in significant improvements in RBC storage quality at 100 μM. Colors representing ranitidine doses correspond to the values detailed in **B**. In **A**–**F**, *y* axes indicate integrated peak areas in AU.

To test whether ranitidine was causally associated and not just correlated with improved energy and redox metabolism in RBCs, and explore the potential of ranitidine as a supplement to improve RBC storage quality as a proof of principle, we incubated human RBCs (*n =* 3) with increasing doses of ranitidine in the presence of 1,2,3-^13^C_3_-glucose for up to 60 minutes (Figure 7A). At increasing doses of ranitidine (Figure 7B) corresponded increasing levels of ^13^C_3_-labeled 1,6-fructose diphosphate (rate-limiting step of glycolysis; plateauing at 100 μM ranitidine; Figure 7C) and ^13^C_3_-lactate (Figure 7D) — suggestive of increased fluxes through glycolysis. RBCs were also obtained from 3 WT and Sphk1-KO mice (34) prior to incubation with 100 μM ranitidine for 60 minutes at 37°C (Figure 7E). After confirming significant decreases in S1P levels in Sphk1-KO mouse RBCs (Figure 7F), we observed that ex vivo incubation with ranitidine promoted increases in the levels of several glycolytic intermediates, including glucose 6-phosphate (G6P), glyceraldehyde 3-phosphate, 2,3-diphosphoglycerate (2,3-DPG), phosphoglycerate, and pyruvate. However, increases in the total levels of lactate were observed only in WT but not in Sphk1-KO mice following incubation with ranitidine. On the other hand, Sphk1-KO mice showed increased steady-state levels of ribose phosphate, suggestive of increased fluxes through the pentose phosphate pathway and altered redox homeostasis (e.g., glutathione, methionine) — consistent with the model in Figure 6D. Finally, the total adenylate pools were increased in WT cells following incubation with ranitidine, but decreased in Sphk1-KO mouse RBCs (Figure 7F). Since different mouse strains respond differently to energy and redox storage lesion (40), we tested storage of RBCs from FVB/J (FVB) mice (*n* = 3; characterized by poor RBC storage quality) and C57BL/6 (B6, *n* = 3; good quality) in the presence of ranitidine (0, 50, 100, or 200 μM) and found a significant improvement in posttranslational recovery, especially at 100 μM (Figure 7G), and increases in RBC S1P levels in both strains (Supplemental Figure 7).

### Proteome integral solubility alteration assay suggests a potential interaction of ranitidine with hemoglobin beta.

**Figure 8:**
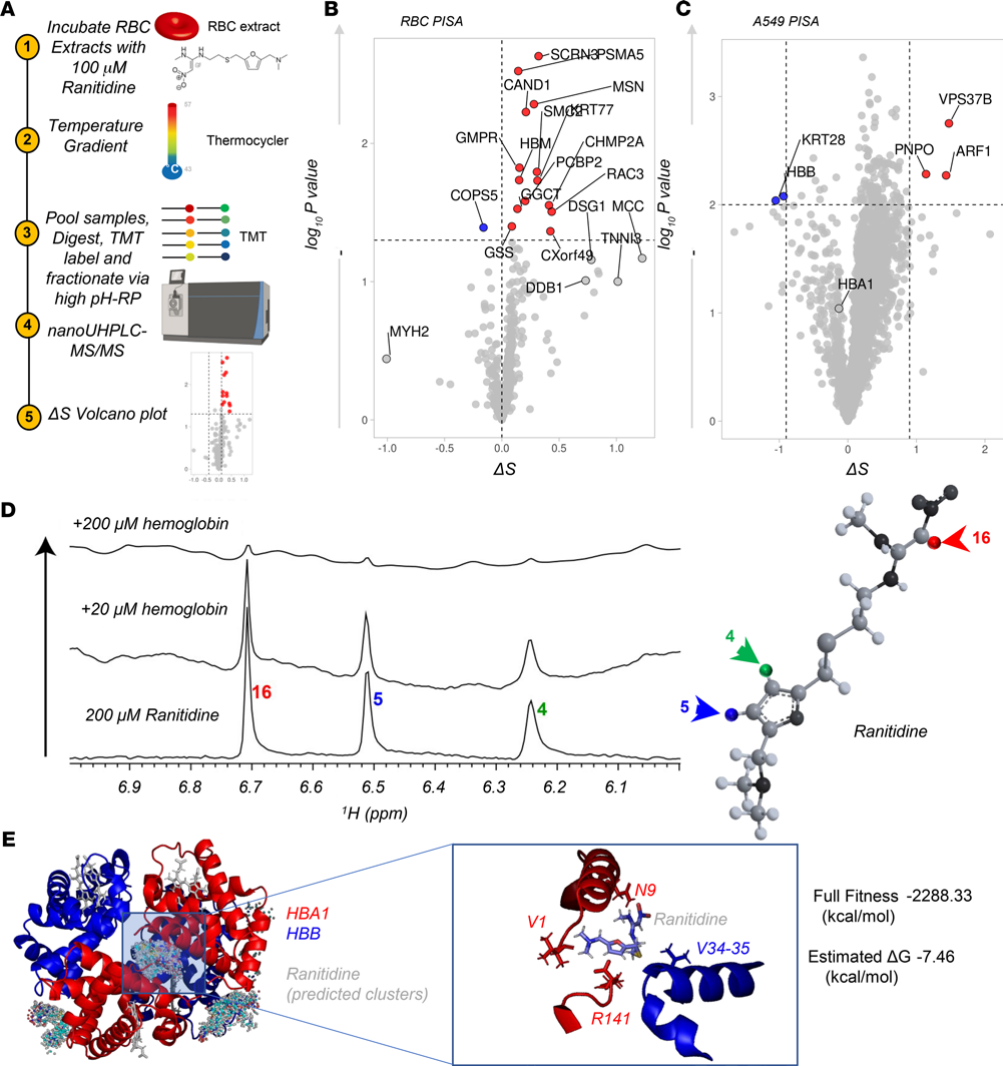
PISA assay in RBCs and A549 epithelial cells in presence of 100 μM ranitidine. (**A**) Overview of the experimental design. (**B** and **C**) Volcano plots show the proteins that are significantly stabilized (red) or destabilized (blue) by ranitidine in RBC and A549 extracts, respectively. (**D**) A 1D ^1^H-NMR spectrum was collected of 200 μM ranitidine alone and in the presence of 20 μM and 200 μM hemoglobin at 25°C. Forty-eight scans were collected on a Varian 900 using the BioPack water sequence implemented with wet water suppression. All spectra are shown at the same vertical scale. The disappearance of peaks means that the resonances of ranitidine, the smaller molecule, is relaxing faster due to binding to the larger molecule, hemoglobin. On the right, an overview of the ranitidine structure with — highlighted in red, blue, green — the proton responsible for the resonances highlighted in the spectra to the left. (**E**) docking of ranitidine with the deoxyhemoglobin tetramer (PDB 1a3n) was calculated in silico with SwissDock. All possible dockings are shown on the left. On the right is a zoom in into the conformation with the lowest estimated Gibbs free energy and best fitness, at the interface between the 2 α-globin chains (at the N-terminus and C-terminus of either HBA1 chain; red).

Finally, to provide a preliminary understanding of the mechanism potentially underlying our observations, we performed proteome integral solubility alteration (PISA) assays to determine ranitidine targets in RBCs (experimental design and volcano plot in Figure 8, A and B, respectively). Interestingly, results identified a small number of potential ranitidine interactors, including hemoglobin subunits (e.g., hemoglobin subunit mu [HBM]) and several enzymes involved in redox homeostasis (e.g., gamma-glutamylcyclotransferase [GGCT], glutathione synthetase [GSS]), GMP metabolism (GMP reductase [GMPR], Ras-related C3 botulinum toxin substrate 3 [RAC3]), structural homeostasis (e.g., moesin [MSN]), vesiculation (secernin 3 [SCRN3]), and protein degradation (Cullin-associated NEDD8-dissociated protein 1 [CAND1], proteasomal subunit A5 [PSMA5]; Figure 8B). However, acknowledging the technical limitations of these experiments in RBCs, owing to the overwhelming abundance of hemoglobins, we decided to repeat the experiment in A549, an epithelial alveolar cell line that has been reported to express hemoglobins (41). As a result (Figure 8C), a cleaner readout was obtained, highlighting a destabilizing effect of ranitidine on hemoglobin beta (HBB) chain. Finally, 1D ^1^H-NMR studies were performed to test the predicted interaction of ranitidine with hemoglobin (Figure 8D). Results indicated the disappearance of peaks assigned to ranitidine protons, suggesting that the resonances of ranitidine, the smaller molecule, were relaxing faster due to binding to the larger molecule, hemoglobin tetramer, in a dose-dependent fashion (0, 20, and 200 μM hemoglobin). We thus used the software SwissDock (42) to perform in silico docking prediction of the interaction between hemoglobin (Protein Data Bank [PDB] 1a3n) and ranitidine. Deoxygenated hemoglobin was selected rather than the oxygenated form because of the impact of ranitidine on S1P observed in this study and prior evidence of S1P interaction with deoxyhemoglobin (35). Results from this computation are shown in Figure 8D — with all the possible docking conformations mapped in the left panel. Zooming into the fitting conformation with the lowest predicted Gibbs free energy (right panel, Figure 8D), ranitidine was predicted to sit at the interface between the N-terminus (V1 and N9) and C-terminus (R141) of the 2 hemoglobin alpha chains, respectively, and in proximity to V34 and V35 of HBB.

## Discussion

In the present study, we provide a comprehensive description of the blood donor exposome — i.e., the compendium of small molecule metabolites that derive from exogenous exposures, including but not limited to diet, xenometabolites (e.g., drugs), habits (e.g., smoking, drinking), and blood processing. Previous studies focused on the metabolic impact of specific exposures, such as donor habits, e.g., smoking (24, 25); consumption of alcohol (43), coffee (44), or taurine (45); or blood processing (e.g., plasticizers, storage additives, leukofiltration; ref. 19). Thus, here we mostly focused on an as-yet-unexplored aspect of RBC storage biology: the presence and potential impact of drugs on the metabolism of stored RBCs.

Since blood donor deferral is limited to a short list of drugs impacting coagulation cascades (e.g., blood thinners) and antibiotics (full list available on the American Association of Blood Banks website; ref. 46), it is expected — but to the best of our knowledge unreported — that routine blood donors would be exposed to over-the-counter or prescription drugs at a rate comparable to that in the general population. Personalized medicine efforts worldwide are increasingly challenging the very concept of “healthy” control subjects in medicine. Here we showed that this caveat holds true in a population of healthy donor volunteers, a demographic group that has progressively aged over the past 2 decades and is thus statistically more likely to consume drugs that impact RBC metabolism and storability (e.g., blood pressure medications, statins, biguanides, aspirin, antacids). From the present analysis, it is not possible to determine (a) whether those drugs were present only in traces or in concentrations of potential relevance to the recipient; (b) how long before the blood donation the drugs had been taken; (c) whether the drugs are enriched in RBCs or in the residual plasma in the supernatants, which accounts for approximately 10% of the storage medium in which the RBCs are resuspended. Some of the drugs, such as sildenafil and progestin (birth control), were detected in donors with a demographic consistent with their clinical target (e.g., men older than 65 years and women of reproductive age, respectively), providing internal validation for the present analysis. These observations were accompanied by the detection of some metabolites of these drugs in the blood units, consistent with the observed decrease in levels of most detected drugs as a function of storage duration in longitudinal samples. While drug metabolites may have ended up in the units upon metabolism from other organs prior to donation (e.g., liver), here we provide preliminary evidence that incubation of leukocyte- and platelet-filtered RBCs with a drug library of 1366 FDA-approved drugs afforded the detection of some drug metabolites, suggestive of active drug metabolism for a subset of small molecule chemicals in the mature erythrocyte. This observation expands on recent reports on the metabolism of antiretrovirals (e.g., tenofovir; ref. 15) and antimalarials (14) in the mature erythrocyte, a phenomenon perhaps explained by the activity of RBC abundant cytochrome *b*_5_ reductase or other diaphorases in this cell (10).

While future studies will further delve into the role of RBCs in drug metabolism, here we leveraged a high-throughput drug screening to confirm that approximately 65.1% of FDA-approved drugs in the library we tested had a significant effect on RBC metabolism. This is relevant in that it supports the hypothesis that drug consumption may represent an unappreciated contributor to the metabolic heterogeneity of stored blood units. Our observation does not necessarily hold a negative connotation, in that some drugs have been observed to favor RBC energy metabolism and decrease oxidant stress markers, including markers of poor storage quality and posttransfusion performance (30, 39, 40). One such drug we focused on here is ranitidine, a histamine H2 receptor antagonist that is a prescribed and over-the-counter drug in the treatment of gastrointestinal diseases related to gastric acid hypersecretion. We show that detection of ranitidine, while rare in the donor population tested here, is associated with increases in energy metabolism and decreased oxidant stress in the REDS-III RBC-Omics recalled donor cohort. These observations were validated in vitro in human RBCs and ex vivo in mouse RBCs, in which ranitidine as a supplement to storage additives promoted increases in the levels of S1P and fluxes through glycolysis in WT mice, but not in mice lacking the expression of Spk1 — the rate-limiting enzyme of S1P synthesis. RBCs from 2 different mouse strains stored in the presence of ranitidine were characterized by higher S1P levels, which normally decrease as a function of storage under blood bank condition; and increased posttransfusion recovery, i.e., the percentage of transfused RBCs that still circulate 24 hours after transfusion. In prior work, we have shown that RBCs upregulate S1P synthesis via Sphk1 to counteract hypoxia at high altitude or in sickle cell disease (34, 35). Though further mechanistic studies will be necessary, overall our data are consistent with two potential mechanisms through which ranitidine may contribute to improving RBC storage quality. First, ranitidine may directly promote S1P synthesis via Sphk1. In turn, by stabilizing the tense deoxygenated state of hemoglobin, S1P promotes the displacement and activation of glycolytic enzymes from the N-terminus of band 3, which favors metabolic fluxes through glycolysis (34, 35). Here we also provide evidence (PISA, in silico docking, and NMR) for a second tentative model: in analogy to S1P, ranitidine may directly interact with hemoglobin.

In conclusion, we provide evidence of high rates of detection of drugs, drug metabolites, and other exposome compounds in stored RBC units. We also show an impact of drugs (65% of 1366 tested) on RBC metabolism and offer an example of the translational clinical relevance of our findings in transfusion medicine. Through a combination of high-throughput metabolomics and machine learning elaborations, the data generated in this study helped to formulate a hypothesis that we further tested mechanistically in a pilot, proof-of-principle study (i.e., ranitidine improves RBC energy and redox metabolism, storage, and posttransfusion performance) and several others that it is hoped will fuel years of follow-up investigations. Despite the proof-of-principle nature of the validation studies on ranitidine as a potential additive, we stress that since the generation of these data, ranitidine was recalled by FDA owing to an ongoing investigation of a contaminant (*N*-nitrosodimethylamine) in some products depending on storage conditions.

## Methods

Details are provided in Supplemental Methods.

### REDS-III RBC-Omics study participants and samples.

Donor selection and recruitment for the RBC-Omics study have been previously detailed (20,47, 48). Samples from 13,403 RBC units were stored for approximately 39–42 days prior to evaluation for osmotic (20, 49) and oxidative hemolysis (20) (12,799 and 10,476, respectively). A subset (*n =* 250) of extreme hemolyzers in this cohort (5th and 95th percentiles) donated a second unit, which was sampled on storage days 10, 23, and 42. A subset of these samples (599 total samples) were made available for metabolomics via ultra-high-pressure liquid chromatography coupled to high-resolution mass spectrometry (UHPLC-MS; Vanquish–Q Exactive, Thermo Fisher Scientific; refs. 20, 49).

### High-throughput drug screening.

Three leukocyte-filtered units from healthy donor volunteers were collected in CP2D-AS-3 and incubated with a screening library of 1366 FDA-approved drugs at 10 μM for 24 hours at 37°C under sterile conditions in 96-well plates. Automated extractions and high-throughput metabolomics analyses are detailed in previous technical reports (50) and in the supplemental material (50, 51).

### Incubation with ranitidine of human and mouse RBCs.

RBCs from blood donors or mice — WT or Sphk1-KO, as described previously (34) — were incubated in CPD-AS-3 or CPDA1, respectively, with increasing doses (10, 25, 50, 100, 200 μM) of ranitidine (MilliporeSigma, 1598405), in the presence of 1,2,3-^13^C_3_-glucose (MilliporeSigma, 720127) to determine fluxes through glycolysis based on the isotopologue M+3 of lactate (8).

### Posttransfusion recovery of mouse RBCs stored in the presence of ranitidine.

Mouse RBCs were obtained by intracardiac puncture from WT FVB and B6 mice (The Jackson Laboratory). All mice were housed in the University of Virginia vivarium. RBCs were stored in CPDA1 additive (40), either untreated or supplemented with 50, 100, and 200 μM ranitidine for up to 8 days at 4°C in at least 3 independent experiments (*n =* 3 per group), prior to determination of posttransfusion recovery (ref. 40 and supplemental material).

### PISA assay.

PISA experiments were performed as described extensively in methodological articles (52, 53) by determining temperature dependency of protein solubility after incubation of human RBCs or A549 epithelial cells (*n =* 5) in the presence or absence of 100 μM ranitidine in the interval 43°C–57°C. Protein fractions underwent tandem mass tag (TMT) labeling, high-pH reversed-phase fractionation, and nano-UHPLC-MS/MS proteomics (Orbitrap Fusion Lumos, Thermo Fisher Scientific).

### 1D nuclear magnetic resonance assay of ranitidine and hemoglobin interaction.

1D ^1^H-NMR spectrum was collected at 25°C for 200 μM ranitidine alone and in the presence of 20 or 200 μM human hemoglobin tetramers (MilliporeSigma, H7379-1G). A total of 48 scans were collected on a Varian 900 using the BioPack water sequence implemented with wet water suppression.

### Statistics.

Graphs and statistical analyses (either *t* test or repeated-measures ANOVA) were prepared with GraphPad Prism 5.0 and MetaboAnalyst 4.0. Drug library screening data were processed via TSNE to highlight the extent of metabolic impact of drug exposure on RBC metabolism and biclustering analysis to identify specific metabolic targets for subsets of drugs. A *P* value less than 0.05 was considered significant.

### Study approval.

Donor selection and recruitment for the RBC-Omics study were performed under approved protocols (BioLINCC Study HLB02071919a), as previously described (47–49). All procedures involving mice were performed under a protocol approved by the IACUC of the University of Virginia, Charlottesville.

## Author contributions

TN, DS, and ADA performed metabolomics analyses. AB, BOP, and ADA performed data analysis. LJD, SK, and MPB performed blood donor studies. AH, AS, YX, and JCZ performed animal studies. AI and KCH performed PISA studies. JSR and EZE performed NMR studies. TN and DS contributed equally to this study, though TN participated in the study design and development of analytical tool, as well as training of DS.

## Supplementary Material

Supplemental data

Supplemental Table 1

## Version 1 12/22/2020

In-Press Preview

## Version 2 02/08/2021

Electronic publication
