# Supplementary material for: Blood donor exposome and impact of common drugs on red blood cell metabolism
Source: JCI Insight. 2021 Feb 8;6(3):e146175. doi: 10.1172/jci.insight.146175 (PMC7934844; doi:10.1172/jci.insight.146175)
Supplement: Supplemental data [file jciinsight-6-146175-s084.pdf]

SUPPLEMENTARY MATERIAL

TABLE OF CONTENTS

SUPPLEMENTARY MATERIALS AND METHODS EXTENDED 2

SUPPLEMENTARY REFERENCES 5

SUPPLEMENTARY FIGURES 6

SUPPLEMENTARY FIGURE 1 6
SUPPLEMENTARY FIGURE 2 7
SUPPLEMENTARY FIGURE 3 8
SUPPLEMENTARY FIGURE 4 9
SUPPLEMENTARY FIGURE 5 10
SUPPLEMENTARY FIGURE 6 11
SUPPLEMENTARY FIGURE 7 12

SUPPLEMENTARY TABLES .... XLSX

Supplementary METHODS – EXTENDED

REDS-III RBC-Omics study participants and samples: Donor selection and recruitment for the RBC-Omics study under approved protocols (BioLINCC Study: HLB02071919a) were previously detailed.[1–3] Donors were enrolled at the four participating REDS-III US blood centers. Overall, 13,758 whole blood donors were enrolled and 13,403 (97%) age 18+ provided informed consent to participate in the study; of these, 12,799 and 10,476 were evaluated for osmotic and oxidative hemolysis, respectively on RBCs stored for ~39-42 days. Extreme hemolyzers (5th and 95th percentile) from the donors tested for end of storage oxidative hemolysis (n = 664) were asked to donate a second unit of blood. These units were sterilely sampled at storage day 10, 23 and 42 for osmotic and oxidative hemolysis. A subset of these samples (599 in total) was analyzed through ultra-high-pressure liquid chromatography coupled to high resolution mass spectrometry (UHPLC-HRMS). Blood collection, sample processing and other aspects of the screening and recall phases of the RBC-Omics Study have been extensively described.[4,5]

Oxidative and osmotic hemolysis: Oxidative hemolysis was determined at the University of Pittsburgh and Vitalant Research Institute as reported and further detailed in references. Briefly, RBCs were incubated with 2,2'-azobis-2-methyl-propanimidamide, dihydrochloride (AAPH, 150mmoL) to determine susceptibility to oxidative hemolysis, as extensively described.[4] Osmotic hemolysis measurements were performed through standard pink tests, as described.[4,5]

Sample processing and metabolite extraction: An isotopically labeled internal standard mixture including a mix of $^{13}C^{15}N$-labeled amino acid standards (2.5 μM) was prepared in methanol. A volume of 100μl of frozen RBC aliquots was mixed with water and the mixture of isotopically labeled internal standards (1:1:1, *v/v/v*). The samples were extracted with methanol (final concentration of 80% methanol). After incubation at −20°C for 1 hour, the supernatants were separated by centrifugation and stored at −80°C until analysis. Samples were vortexed[6] and insoluble material pelleted as described.[7,8]

Ultra-High-Pressure Liquid Chromatography-Mass Spectrometry metabolomics: Analyses were performed using a Vanquish UHPLC coupled online to a Q Exactive mass spectrometer (Thermo Fisher, Bremen, Germany). Samples were analyzed using a 3 minute isocratic condition[9] or a 5, 9 and 17 min gradient as described.[8,10] Solvents were supplemented with 0.1% formic acid for positive mode runs and 1 mM ammonium acetate for negative mode

runs. MS acquisition, data analysis and elaboration was performed as described.[8,9] Additional analyses, including untargeted analyses and Fish score calculation via MS/MS, were calculated against the ChemSpider database with Compound Discoverer 2.0 (Thermo Fisher, Bremen, Germany).

High-throughput drug screening: Three leukocyte-filtered units from healthy donor volunteers were collected in CP2D-AS-3 and incubated with a screening library of 1,366 Food and Drug Administration-approved drugs at 10 uM concentrations (upon dilution 1:1:000 of 10 mM original stocks – chemical details about the library are extensively provided in **Supplementary Table 1**) for 24h at 37°C under sterile conditions in 96 well plates. Automated extractions were performed on 96 well-plate format by adding 100 ul of ice-cold extraction solution (methanol:acetonitrile:water 5:3:2 v/v) through automated liquid handler (OpenTron). Cell lysates were transferred via 96 well-plate 0.1 um filters through positive pressure (25 bar) with nitrogen gas. Lysates were thus transferred via liquid handlers into separate plates prior to cooled dry down under vacuum (96-well compatible) and resuspended in ddH_2O + 0.1% formic acid or ddH_2O + 10 mM ammonium acetate prior to high-throughput metabolomics screening of 16 plates per polarity in positive and negative ion modes with a previously described 5 min high-throughput gradient-based method, respectively. The last column of each plate contained 5 control replicates (no drug) and tech mixes (pool of 5 ul of sample extracts from each plate), which were used for intra- and inter-plate normalization purposes post-hoc via MetaboDrift. Samples were run in randomized order through a Vanquish UHPLC system (Thermo Fisher) updated with a 12-plate charger, coupled to a high-resolution quadrupole orbitrap mass spectrometer (QExactive), operated as described.[11,12]

Incubation with ranitidine of Human and Mouse RBCs: RBCs from human or mice – either WT or Sphk1 KO, as described[13] were incubated in CPD-AS-3 or CPDA1, respectively, with increasing doses (10, 25, 50, 100, 200 uM) of ranitidine (product no: 1598405 – Millipore Sigma), in presence of 1,2,3-$^{13}C_3$-glucose (product no: 720127 - SIGMA Aldrich, St Louis, MO, USA). Tracing experiments were performed as described,[14] to determine fluxes through glycolysis based on the isotopologue +3 of lactate.

Post-transfusion recovery of mouse RBCs stored in presence of ranitidine: Mouse RBCs were obtained by intracardiac puncture from wild-typeFVB/J and C57BL6/J mice (The Jackson Laboratory). All mice were housed in the University of Virginia vivarium, and all procedures were

performed under an Institutional Animal Care and Use Committee–approved protocol. RBCs were stored as previously described,[15] in CPDA1 additive, either untreated or supplemented with 50, 100, 200 uM ranitidine for up to 8 days at 4ºC in at least three independent experiments (n=3 per group). At the end of the storage period, RBCs were transfused into B6 x Ubi-GFP+ mice. Prior to transfusion, fresh HOD^+ RBCs were added to the stored FVB RBCs and the mixture of RBCs was transfused as described.[15] Post-transfusion recoveries (PTR) were determined by sampling peripheral blood at 24 hours post-transfusion and enumerating stored FVB RBCs (HOD-GFP-) as a ratio of the fresh tracer population (HOD+GFP-) and correcting for pretransfusion ratios.

Proteome Integral Solubility Alteration (PISA) assay: PISA experiments were performed as described extensively in methodological papers,[16,17] by incubating human RBCs or A549 epithelial cells (n=5) in presence or absence of ranitidine 100 uM. Briefly, temperature-dependency of protein solubility in presence or absence of ranitidine was tested in five biological replicates per group in the interval of 43-57ºC, prior to TMT-labeling, high-pH reversed phase fractionation and nanoUHPLC-MS/MS proteomics (Orbitrap Fusion Lumos, Thermo Fisher, Bremen, Germany).

Statistical Analyses: Graphs and statistical analyses (either t-test or repeated measures ANOVA) were prepared with GraphPad Prism 5.0 (GraphPad Software, Inc, La Jolla, CA). High-throughput drug screening data were processed via t-distributed stochastic neighbor embedding (TsNE) to highlight the extent of metabolic impact of drug exposure on red cell metabolism and biclustering analysis to identify specific metabolic targets for subset of drugs in the high-throughput screening.

1D Nuclear Magnetic Resonance assay of ranitidine and hemoglobin interaction

1D NMR 1H spectrum was collected of 200 uM ranitidine alone and in the presence of 20 uM human hemoglobin tetramers (product no: H7379-1G – SIGMA Aldrich, St Louis, MO, USA) and 200 uM hemoglobin at 25ºC. A total of 48 scans were collected on a Varian 900 using the BioPack water sequence implemented with wet water suppression.

Supplementary REFERENCES

1. Kanias T, Lanteri MC, Page GP, Guo Y, Endres SM, Stone M, et al. Ethnicity, sex, and age are determinants of red blood cell storage and stress hemolysis: results of the REDS-III RBC-Omics study. Blood Adv. 2017 Jun 27;1(15):1132–41.
2. Stone M, Keating SM, Kanias T, Lanteri MC, Lebedeva M, Sinchar D, et al. Piloting and implementation of quality assessment and quality control procedures in RBC-Omics: a large multi-center study of red blood cell hemolysis during storage. Transfusion . 2019 Jan;59(1):57–66.
3. Lanteri MC, Kanias T, Keating S, Stone M, Guo Y, Page GP, et al. Intradonor reproducibility and changes in hemolytic variables during red blood cell storage: results of recall phase of the REDS-III RBC-Omics study. Transfusion . 2019 Jan;59(1):79–88.
4. Kanias T, Lanteri MC, Page GP, Guo Y, Endres SM, Stone M, et al. Ethnicity, sex, and age are determinants of red blood cell storage and stress hemolysis: results of the REDS-III RBC-Omics study. Blood Adv. 2017 Jun 23;1(15):1132–41.
5. Endres-Dighe SM, Guo Y, Kanias T, Lanteri M, Stone M, Spencer B, et al. Blood, sweat, and tears: Red Blood Cell-Omics study objectives, design, and recruitment activities. Transfusion . 2018 Sep 28;
6. Reisz JA, Nemkov T, Dzieciatkowska M, Culp-Hill R, Stefanoni D, Hill RC, et al. Methylation of protein aspartates and deamidated asparagines as a function of blood bank storage and oxidative stress in human red blood cells. Transfusion . 2018 Dec;58(12):2978–91.
7. Nemkov T, Hansen KC, Dumont LJ, D'Alessandro A. Metabolomics in transfusion medicine. Transfusion . 2016 Apr;56(4):980–93.
8. D'Alessandro A, Nemkov T, Yoshida T, Bordbar A, Palsson BO, Hansen KC. Citrate metabolism in red blood cells stored in additive solution-3. Transfusion . 2017 Feb;57(2):325–36.
9. Nemkov T, Hansen KC, D'Alessandro A. A three-minute method for high-throughput quantitative metabolomics and quantitative tracing experiments of central carbon and nitrogen pathways. Rapid Commun Mass Spectrom RCM. 2017 Apr 30;31(8):663–73.
10. Fu X, Felcyn JR, Odem-Davis K, Zimring JC. Bioactive lipids accumulate in stored red blood cells despite leukoreduction: a targeted metabolomics study. Transfusion . 2016 Oct;56(10):2560–70.
11. Reisz JA, Zheng C, D'Alessandro A, Nemkov T. Untargeted and Semi-targeted Lipid Analysis of Biological Samples Using Mass Spectrometry-Based Metabolomics. Methods Mol Biol Clifton NJ. 2019;1978:121–35.
12. Nemkov T, Reisz JA, Gehrke S, Hansen KC, D'Alessandro A. High-Throughput Metabolomics: Isocratic and Gradient Mass Spectrometry-Based Methods. Methods Mol Biol Clifton NJ. 2019;1978:13–26.
13. Sun K, Zhang Y, D'Alessandro A, Nemkov T, Song A, Wu H, et al. Sphingosine-1-phosphate promotes erythrocyte glycolysis and oxygen release for adaptation to high-altitude hypoxia. Nat Commun. 2016 15;7:12086.
14. Reisz JA, Wither MJ, Dzieciatkowska M, Nemkov T, Issaian A, Yoshida T, et al. Oxidative modifications of glyceraldehyde 3-phosphate dehydrogenase regulate metabolic reprogramming of stored red blood cells. Blood. 2016 22;128(12):e32-42.
15. Howie HL, Hay AM, de Wolski K, Waterman H, Lebedev J, Fu X, et al. Differences in Steap3 expression are a mechanism of genetic variation of RBC storage and oxidative damage in mice. Blood Adv. 2019 Aug 13;3(15):2272–85.
16. Gaetani M, Sabatier P, Saei AA, Beusch CM, Yang Z, Lundström SL, et al. Proteome Integral Solubility Alteration: A High-Throughput Proteomics Assay for Target Deconvolution. J Proteome Res. 2019 01;18(11):4027–37.
17. Li J, Van Vranken JG, Paulo JA, Huttlin EL, Gygi SP. Selection of Heating Temperatures Improves the Sensitivity of the Proteome Integral Solubility Alteration Assay. J Proteome Res. 2020 May 1;19(5):2159–66.

SUPPLEMENTARY FIGURES

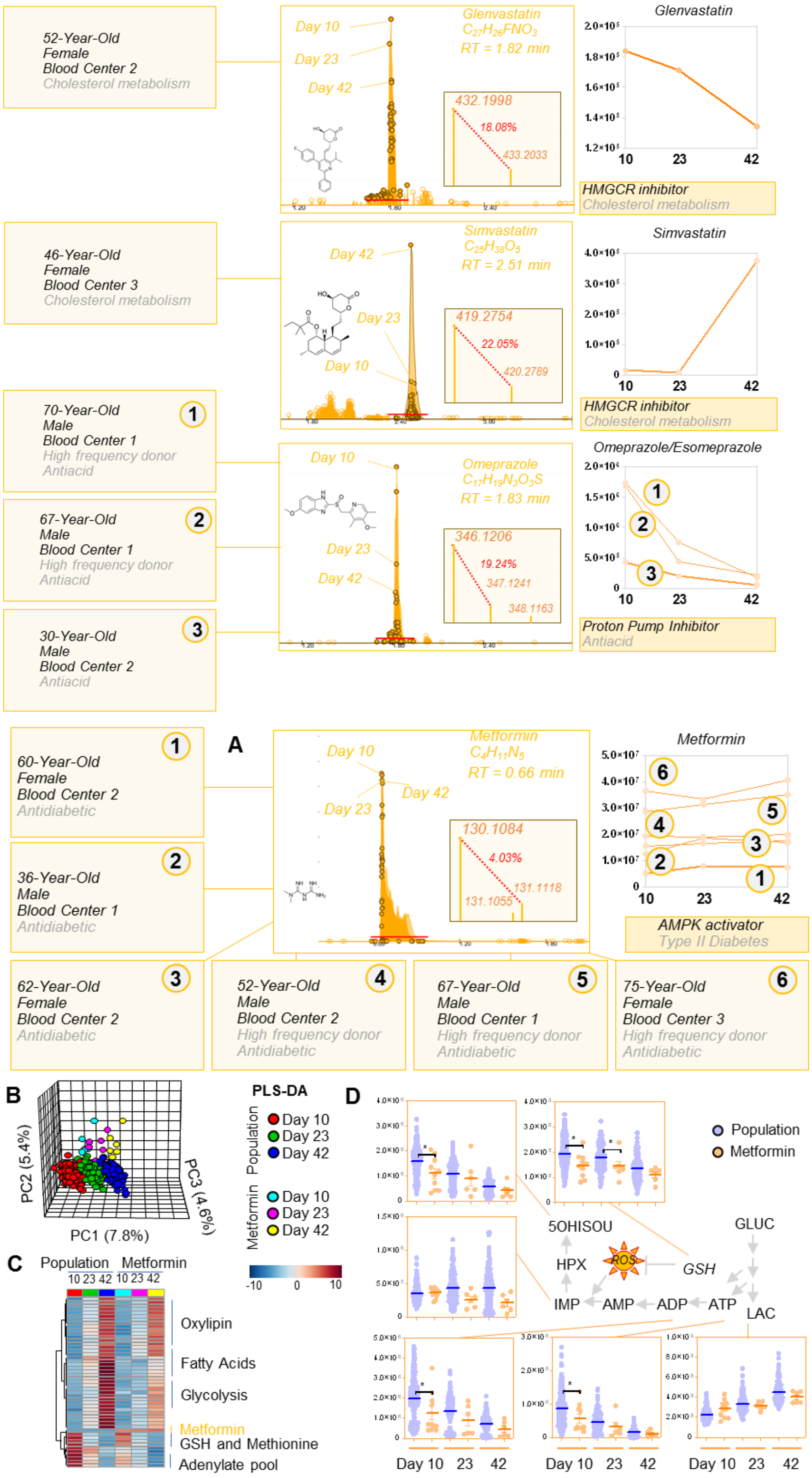

Supplementary Figure 1 – Statins in RBCs from a subset of REDS III RBC Omics donors. Statins (cholesterol metabolism) were detected in RBCs from several healthy donor volunteers. Case studies are described in which the drugs were detected. For each metabolite we provide the original Extract Ion Chromatograms on the background of the whole population (599 samples), molecular formulae, retention times, mass spectra, ^{13}C abundance as percentage of the parent m/z peak, and time course levels of these metabolites (Top half of the figure). In the bottom half, panel **A** highlights the levels of metformin, detected in 6 REDS III volunteers. Since metformin is a drug that impacts cellular metabolism, we identified an impact of metformin on RBC metabolism as gleaned by partial least square-discriminant analysis (**B**) and hierarchical clustering analysis (**C**). A few representative metabolites for metformin positive (orange) RBCs are shown in **D**, against the rest of the population (blue) at storage day 10, 23 and 42.

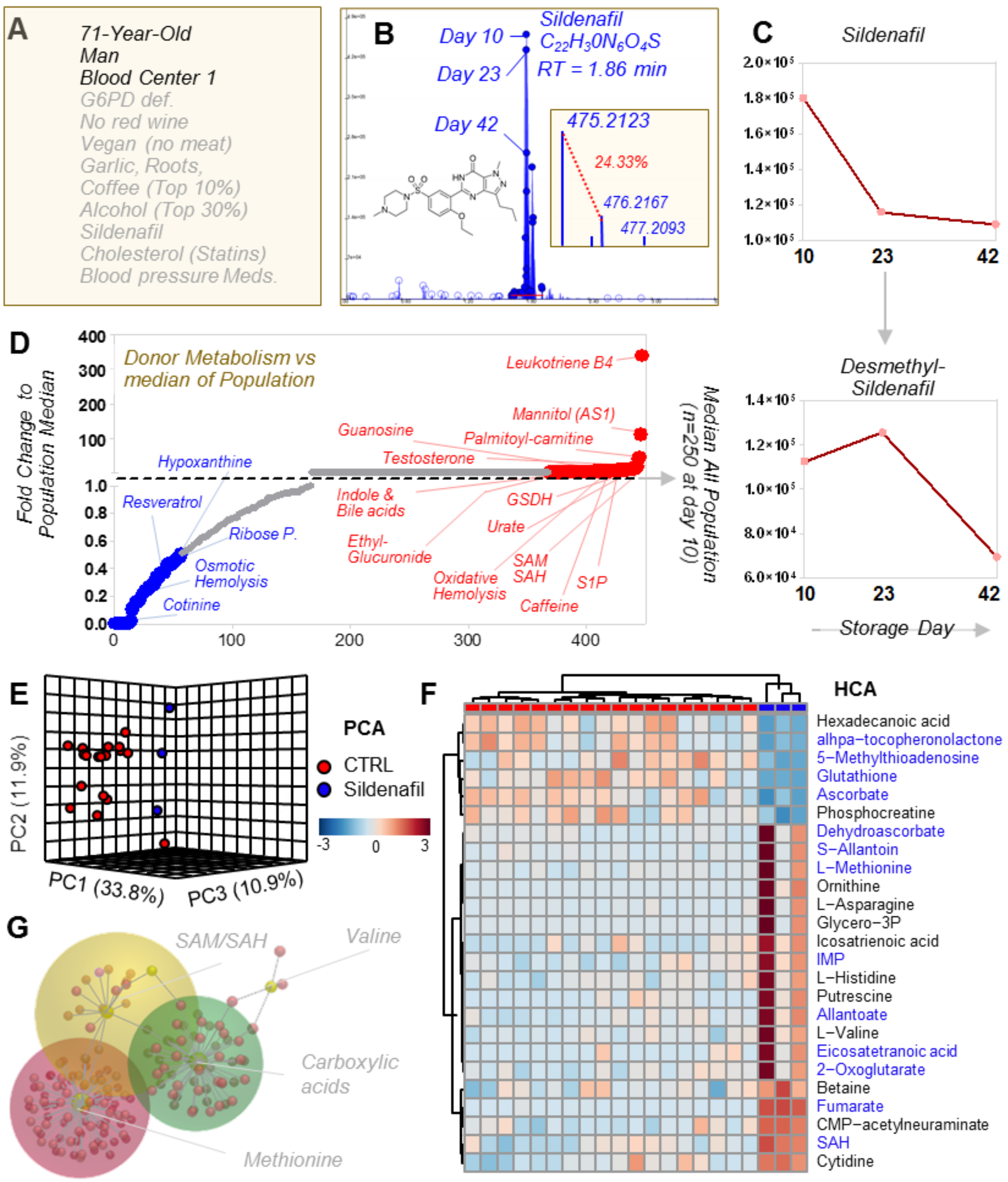

Supplementary Figure 2 – A volunteer was found to be positive for traces of sildenafil (A). In **B**, we provide the original Extract Ion Chromatogram for sildenafil on the background of the whole population (599 samples), its molecular formula, retention time, mass spectra, ^{13}C abundance as percentage of the parent m/z peak (**B**), and time course levels of sildenafil and its bioactive metabolite, desmethylsildenafil (**C**). In **D**, the Manhattan plot shows metabolite levels in this subject as fold-change vs the median of the rest of the population, highlighting a potential metabolic impact of sildenafil (at the net of the influence of other covariates) in the RBCs from this subject, especially with respect to oxidative stress markers. Principal Component Analysis (PCA – **E**), hierarchical clustering analysis (**F**) and pathway analysis (**G**) were used to confirm that sildenafil can impact RBC metabolism – as gleaned from the high-throughput drug screening (i.e., 24h incubation of RBCs – n=3 – to sildenafil at 37°C)

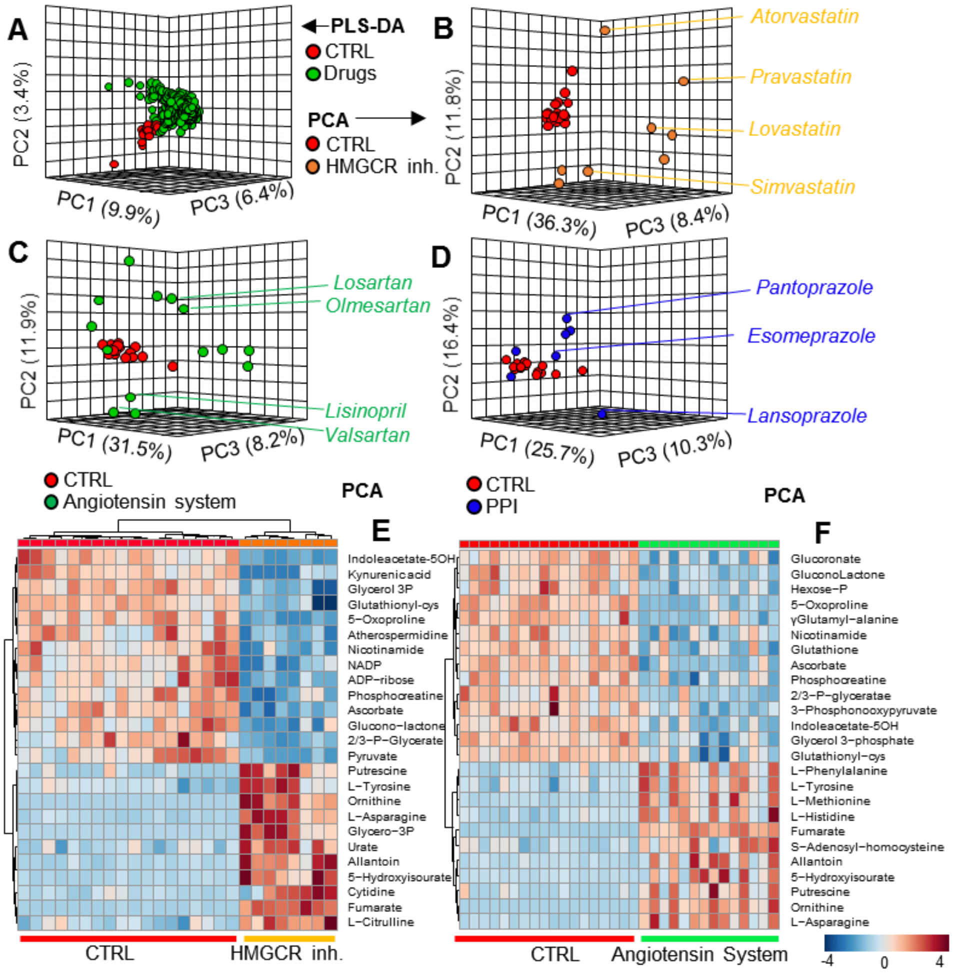

Supplementary Figure 3 – RBC metabolic impact of blood pressure, cholesterol and antiacid drugs in the high-throughput screening. Drugs significantly impacted RBC metabolism (**A**) as gleaned by partial least square-discriminant analyses (PLS-DA - **A**). In **B-D**, PLS-DAs of statins, sartans and proton pump inhibitors (PPI) vs untreated controls. The top 25 significant (T-Test) metabolites whose levels are affected by drugs targeting the angiotensin system (green - **E**) or PPI (blue - **F**) vs control RBCs (red) include amino acid, redox homeostasis-related metabolites (glutathione, pentose phosphate pathway, S-adenoysl-methionine), purines, carboxylic acids.

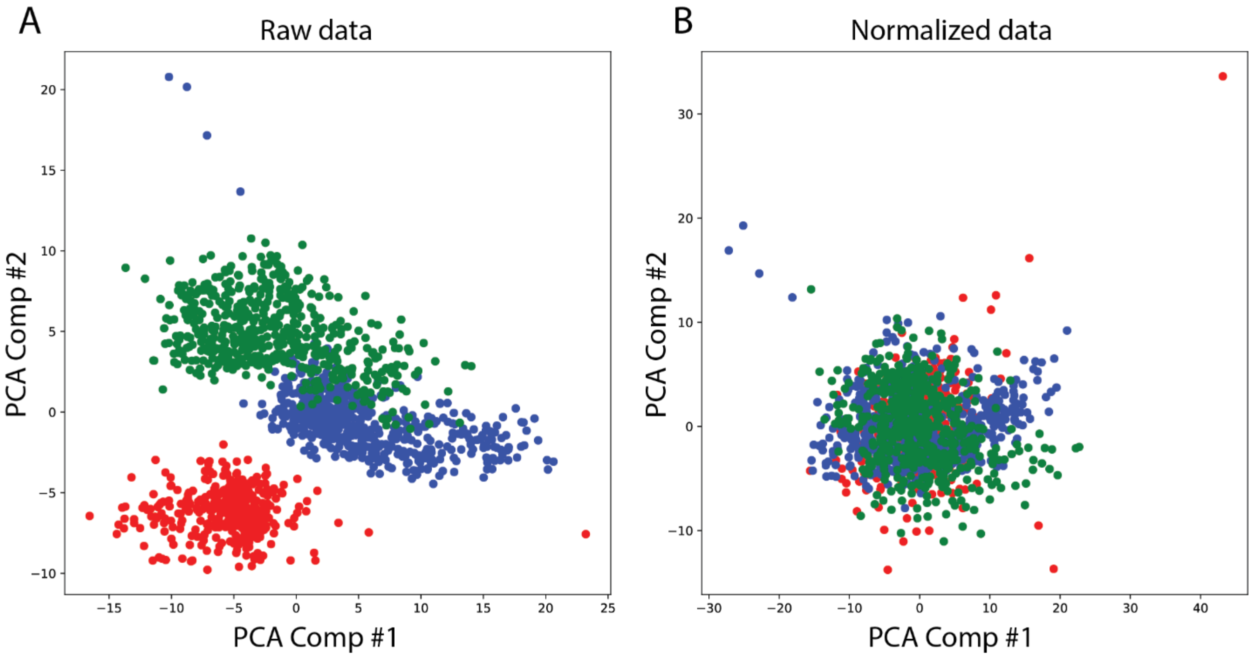

Supplementary Figure 4 – Principal Component Analysis of Metabolomics data from the high-throughput screening of 1,366 FDA approved drugs. Data are shown before (**A**) and after (**B**) normalization. Since samples were distributed over 17 96-well plate and processed over the course of 14 consecutive instrument time-days, batch to batch normalization was performed based on internal controls and vehicle samples that were kept consistent across every plate. Raw and normalized data are available as csv file upon request and will be uploaded on the RBC Atlas - MIRAGES project website - https://www.dalessandrolab.com/mirages-rbc-atlas.

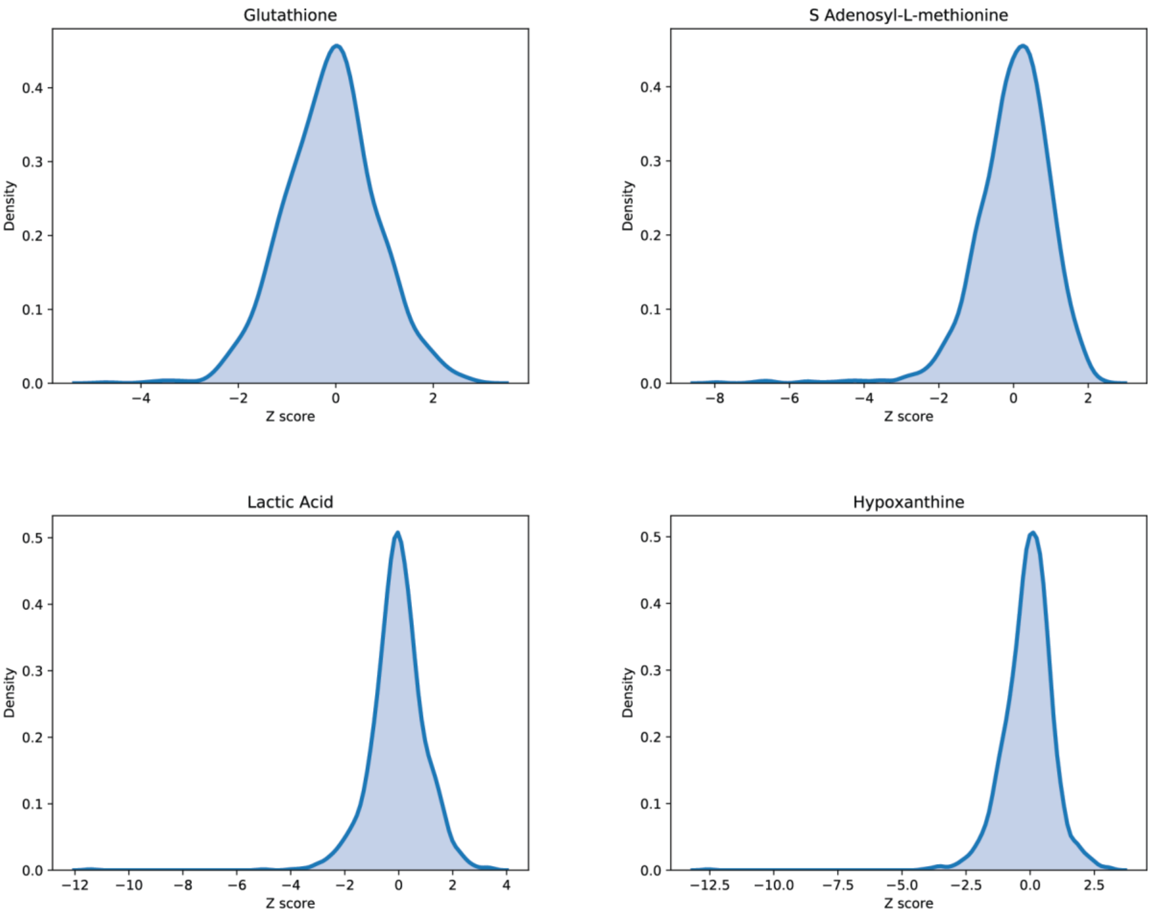

Supplementary Figure 5 - Small molecules have effects to erythrocyte metabolites that have been implicated in several pathologies and storage related changes during including Glutathione, Lactic acid, S-Adenosyl-L-methionine, and Hypoxanthine. Notable drugs are shown as examples of perturbation.

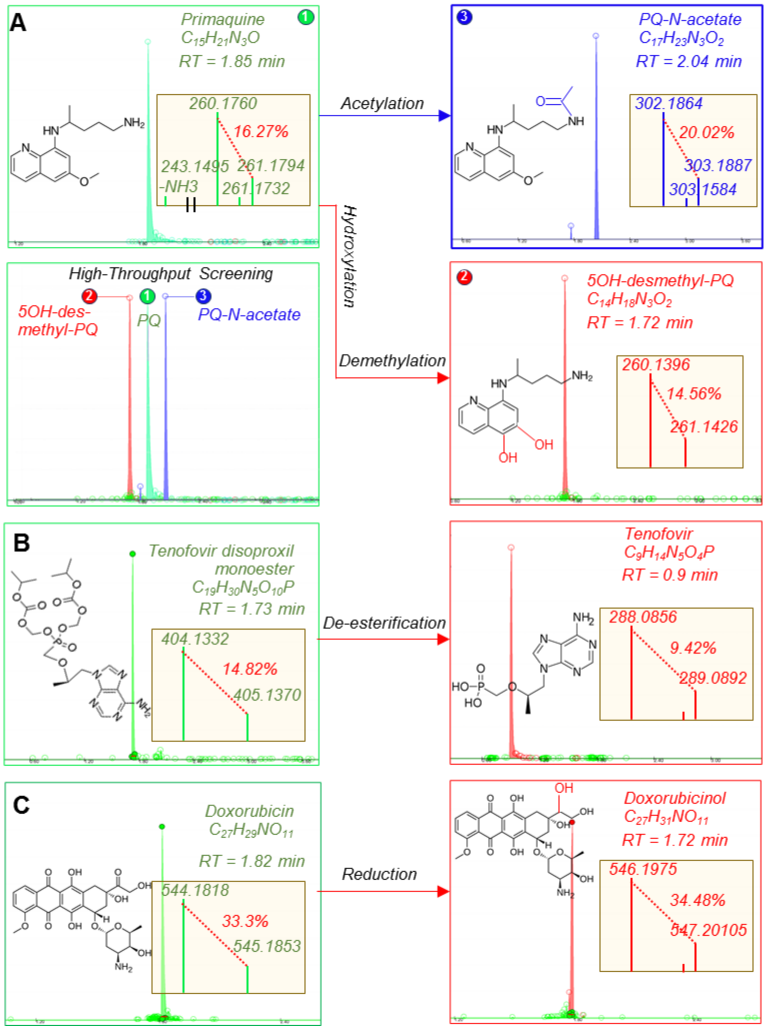

Supplementary Figure 6 - RBC can metabolize a subset of the 1366 drugs they were incubated with in the high-throughput screening, including primaquine to primaquine N-acetate and 5-hydroxy-desemthyl-primaquine (active metabolite – **A**, consistent with previous reports in the literature); tenofovir disoproxil to tenofovir metabolites (**B**) and doxorubicin to doxorubicinol (**C**). For each metabolite we provide the original Extract Ion Chromatograms on the background of the whole population (599 samples), molecular formulae, retention times, mass spectra, ^{13}C abundance as percentage of the parent m/z peak, and time course levels of these metabolites.

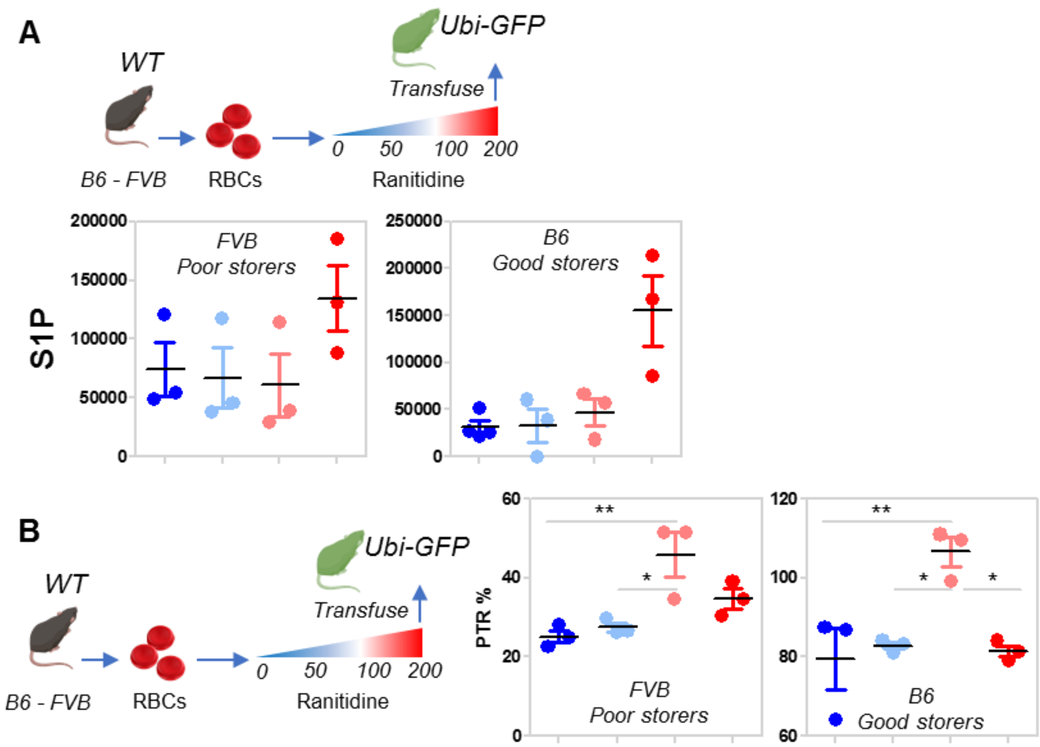

Supplementary Figure 7 – Storage of mouse RBCs with ranitidine increases S1P levels (A) and improves end of storage post-transfusion recovery in poor storing mice (FVB).

RBC-OMICS STUDY GROUP MEMBERS

The NHLBI Recipient Epidemiology Donor Evaluation Study-III (REDS-III), Red Blood Cell (RBC)-Omics Study, is the responsibility of the following persons: Hubs: A. E. Mast, J. L. Gottschall, W. Bialkowski, L. Anderson, J. Miller, A. Hall, Z. Udee, and V. Johnson, BloodCenter of Wisconsin, Milwaukee, WI; D. J. Triulzi, J. E. Kiss, and P. A. D'Andrea, The Institute for Transfusion Medicine (ITXM), Pittsburgh, PA; E. L. Murphy and A. M. Guiltinan, University of California, San Francisco, San Francisco, CA; R. G. Cable, B. R. Spencer, and S. T. Johnson, American Red Cross Blood Services, Farmington, CT; Data coordinating center: D. J. Brambilla, M. T. Sullivan, S. M. Endres, G. P. Page, Y. Guo, N. Haywood, D. Ringer, and B. C. Siege, RTI International, Rockville, MD; Central and testing laboratories: M. P. Busch, M. C. Lanteri, M. Stone, and S. Keating, Blood Systems Research Institute, San Francisco, CA; T. Kanias and M. Gladwin, Pittsburgh Heart, Lung, Blood, and Vascular Medicine Institute, Division of Pulmonary, Allergy and Critical Care Medicine, University of Pittsburgh, Pittsburgh, PA; Steering committee chairman: S. H. Kleinman, University of British Columbia, Victoria, BC, Canada; National Heart, Lung, and Blood Institute, National Institutes of Health: S. A. Glynn, K. B. Malkin, and A. M. Cristman
